# To Prompt or Not to Prompt? A Microrandomized Trial of Time-Varying Push Notifications to Increase Proximal Engagement With a Mobile Health App

**DOI:** 10.2196/10123

**Published:** 2018-11-29

**Authors:** Niranjan Bidargaddi, Daniel Almirall, Susan Murphy, Inbal Nahum-Shani, Michael Kovalcik, Timothy Pituch, Haitham Maaieh, Victor Strecher

**Affiliations:** 1 Personal Health Informatics, College of Medicine & Public Health Adelaide Australia; 2 Insitute for Social Research Michigan University Ann Arbor, MI United States; 3 Department of Statistics Harvard University Boston, MA United States; 4 Jool Health Ann Arbor, MI United States; 5 School of Public Health University of Michigan Ann Arbor, MI United States

**Keywords:** mobile applications, smartphone, self report, health promotion, lifestyle, ubiquitous computing, push notification

## Abstract

**Background:**

Mobile health (mHealth) apps provide an opportunity for easy, just-in-time access to health promotion and self-management support. However, poor user engagement with these apps remains a significant unresolved challenge.

**Objective:**

This study aimed to assess the effect of sending versus not sending a push notification containing a contextually tailored health message on proximal engagement, measured here as self-monitoring via the app. Secondary aims were to examine whether this effect varies by the number of weeks enrolled in the program or by weekday versus weekend. An exploratory aim was to describe how the effect on proximal engagement differs between weekday versus weekend by the time of day.

**Methods:**

The study analyzes the causal effects of push notifications on proximal engagement in 1255 users of a commercial workplace well-being intervention app over 89 days. The app employs a microrandomized trial (MRT) design to send push notifications. At 1 of 6 times per day (8:30 am, 12:30 pm, 5:30 pm, 6:30 pm, 7:30 pm, and 8:30 pm; selected randomly), available users were randomized with equal probability to be sent or not sent a push notification containing a tailored health message. The primary outcome of interest was whether the user self-monitored behaviors and feelings at some time during the next 24 hours via the app. A generalization of log-linear regression analysis, adapted for use with data arising from an MRT, was used to examine the effect of sending a push notification versus not sending a push notification on the probability of engagement over the next 24 hours.

**Results:**

Users were estimated to be 3.9% more likely to engage with the app in the next 24 hours when a tailored health message was sent versus when it was not sent (risk ratio 1.039; 95% CI 1.01 to 1.08; *P*<.05). The effect of sending the message attenuated over the course of the study, but this effect was not statistically significant (*P*=.84). The effect of sending the message was greater on weekends than on weekdays, but the difference between these effects was not statistically significant (*P*=.18). When sent a tailored health message on weekends, the users were 8.7% more likely to engage with the app (95% CI 1.01 to 1.17), whereas on weekdays, the users were 2.5% more likely to engage with the app (95% CI 0.98 to 1.07). The effect of sending a tailored health message was greatest at 12:30 pm on weekends, when the users were 11.8% more likely to engage (90% CI 1.02 to 1.13).

**Conclusions:**

Sending a push notification containing a tailored health message was associated with greater engagement in an mHealth app. Results suggested that users are more likely to engage with the app within 24 hours when push notifications are sent at mid-day on weekends.

## Introduction

### Background

Evidence demonstrates that providing engaged individuals with a digital behavior change intervention improves health outcomes [1-3]. In recent years, owing to increasingly ubiquitous smartphone ownership at a population level and rapidly reducing digital divides [4], smartphone apps have emerged as an important channel for health behavior interventions. As individuals are in the vicinity of their smartphone most of the time, smartphone apps can be used to sense users’ everyday context and offer behavioral interventions at the most suitable moments. Unlike the traditional health care system, most mobile apps are inexpensive and easy to access on demand. They overcome demographic, socioeconomic, and geographical barriers to access by being able to reach populations that were unreachable until recently [5]. Consequently, smartphone health apps are now widely recognized as a public health promotion resource. For example, the World Health Organization Mental Health Action Plan 2013-2020 recommends “the promotion of self-care, for instance, through the use of electronic and mobile health technologies” [6]. Websites of prominent and large public health organizations (such as the UK National Health Service [NHS] website NHS Choices or Reachout Australia [7]) have also begun formally endorsing and recommending effective apps.

A pressing concern for the field of mobile health (mHealth), however, is the high rate of disengagement among individuals who choose to install an app. After installing a mobile app, over 80% of app users use it only once and eventually delete it [8]. Only 5% of the apps continue to be used beyond a month [9]. Even among those who use the apps, the amount of use depends on an individual’s health and behavioral characteristics [10-12].

The lack of participant engagement is not unique to mHealth apps. In fact, traditional Web-based interventions observe decreases in engagement over time, with large proportions of participants dropping out or discontinuing the use of the app completely [13]. Although when compared with traditional internet interventions, the more recent smartphone app interventions have the potential to reach participants at moments when they are most likely to engage. It is disconcerting that similar underlying disengagement patterns are emerging even with more advanced technology. Consequently, despite the potential of smartphone apps, lack of participant engagement with apps, which is critical to the success of interventions focused on health behavior change, is of major concern. Given the fast pace of smartphone health app development, there is a pressing need for novel research to enhance engagement with these apps.

Effective engagement with digital health apps is particularly critical when this engagement is part of the health intervention, as opposed to simply opening the app. For example, engagement might be classified as use of the app to self-monitor health behaviors and feelings; self-monitoring has been demonstrated to have a positive impact on well-being and improve health behaviors [14,15]. Engagement could also be the subjective quality of user experiences with the app, which can be influenced by design elements [16]. Individual characteristics such as age, education levels, and state of health are known to equally influence the adoption of mHealth apps, in addition to factors such as being female, younger, higher education levels, and lower body mass index increasing the odds [17,18]. Another factor affecting the dropout in mobile app use could be the timing of intervention offered. For example, participants in an app study reported they were likely to drop out if the intervention did not meet their expectations and needs at the right time [19]. On the other hand, some qualitative research suggests that individuals tend to use app-based interventions in brief bursts only, often when needed and in a fleeting manner [20,21]. In this context, it could be argued that even short-term or varying intensity patterns of use with app-based behavior change interventions could be beneficial to users. For example, in the case of a self-monitoring app intervention, some individuals might monitor more frequently than others, but irrespective of how often they engage, every act of self-monitoring enables users to learn some strategies that can be practiced to improve behaviors without additional guidance from the intervention. Thus, every time a user opens and interacts with the intervention activity of the app, they are effectively engaged.

As mobile phones are usually switched on and nearly always with users, the majority of app-based interventions adopt prompting as a strategy to encourage interaction and engagement. In apps, prompts are implemented as push notifications, which appear on the smartphone screen at a programmed time. Both the content and timing of a push notification are programmable. Consequently, smartphone users receive a deluge of push notifications daily, approximately 50 time-varying notifications, from a diverse set of apps [22]. This sheer volume of notifications throughout the day has the potential to further exacerbate disengagement with the apps. A few studies that observed how users responded to push notifications received on their smartphone over the course of a day suggest that users are most likely to ignore a vast majority of notifications even when the notifications come from apps of importance to them [23,24]. There are also concerns that receiving too many notifications might increase users’ inattention and reduce well-being [25].

Due to advances in smartphone-sensing capabilities along with algorithmic advances, apps can now utilize users’ context, for example, location, social setting, and activity level to determine the most opportune times to send push notifications. Studies suggest that users are receptive to notification interruptions at convenient times [26-28]. Within the human-computer interaction field, *interruptibility research* has emerged, with many studies focused on understanding and anticipating suitable moments for interruption [29-32]. However, most studies concerning the effects of push notification interruptions on engagement are observational studies based on small convenience samples with neither randomization nor control conditions. Thus, although rapid advances are being made in devising algorithms to predict when to interrupt users, because of the lack of appropriately designed studies, there is a significant gap in knowledge concerning when particular types of interruptions are effective.

In contrast, the fields of public health and psychology contain several well-designed randomized controlled trial studies to evaluate text-messaging interventions that are similar to push notification interventions in mHealth apps [33,34]. Furthermore, a recent study randomized participants into 3 different conditions based on the different approaches used to determine the timing of push notification [35]. These studies, using baseline randomization of individuals into different conditions, were designed to compare between conditions in terms of a distal outcome (such as the percentage of notifications viewed or overall usage). However, baseline randomization is not suitable for addressing questions comparing prompts at different times or comparing a prompt (vs no prompt) in terms of their effect on near time, proximal engagement, and the conditions in which a prompt would be more or less beneficial. Indeed, on any given day, users may be concerned with the contingencies and demands of the day so that even a self-determined user may not think or remember to access support on the app.

To our knowledge, this is the first study focusing on the effects of push notifications containing contextually tailored health messages on near-time, proximal engagement with the app. Here, engagement in response to a prompt is operationalized as the user completing the self-monitoring intervention activity in the app within 24 hours of receiving the push notification (as opposed to just opening the app). This study examines data collected from a microrandomized trial (MRT) [36] implemented within the JOOL app, a commercial workplace well-being intervention product [37]. In an MRT, each user is randomized multiple times over a period of weeks and months. In the JOOL app, push notifications may be randomized at each of 6 time points per day. This repeat-randomization design adjusts for potential biases that might arise from within and between the individual factors. For instance, randomizing the decision to send a push notification at a time point ensures that within- and between-user factors contributing to day-to-day variations in engagement are balanced approximately evenly across both push notification and no push notification conditions. The specific primary and secondary aims and hypotheses of this study are presented in the next section.

### Aims and Hypotheses

The specific primary and secondary aims and hypotheses are as follows:

#### Primary Aim

To test whether sending a push notification containing a contextually tailored health message versus not sending push notification (in moments of availability) results in an increased likelihood of proximal engagement with the app (ie, the user is self-monitored via the app at some point in the next 24 hours).

#### Hypothesis

We hypothesize that on an average, sending a push notification containing a contextually tailored message will lead to a greater likelihood of proximal engagement with the app as compared with not sending the push notification.

#### Secondary Aim 1

To test whether the effect of sending a push notification containing a tailored message (vs not sending a push notification) on the likelihood of proximal engagement with the app differs by week in the study (there are approximately 12 weeks in the study).

#### Hypothesis

We hypothesize that the effect of sending a push notification containing a tailored message will differ by week in the study. Specifically, we hypothesize that the effect will be greater earlier in the study than later in the study.

#### Secondary Aim 2

To test whether the effect of sending a push notification containing a tailored message (vs not sending a push notification) on the likelihood of proximal engagement with the app differs by weekdays (Monday to Friday) versus weekends (Saturday or Sunday).

#### Hypothesis

We hypothesize that the effect of sending a push notification containing a tailored message will differ by weekdays versus weekends. Specifically, we hypothesize that the effect will be greater when the push notification is on weekends than on weekdays.

The exploratory aim focused on whether the effect of sending a push notification containing a tailored message (vs not sending a push notification) on the likelihood of proximal engagement with the app differs by the time of day within weekday-versus-weekend.

For all aims, the effect of sending a push notification is defined more precisely as the effect of sending a push notification (and not sending a subsequent push notification over the next 24 hours) versus not sending a push notification now or over the next 24 hours.

## Methods

### Intervention and Push Notification

The JOOL app is a smartphone-based behavioral intervention using self-monitoring and feedback strategies to help users find their purpose in life and develop the energy and willpower they need to live in accordance with their purpose every day. To engage in self-monitoring activity, users open the app and record on a scale of 0 (worse) to 100 (best) values for daily energy, willpower, sleep, presence, physical activity, creativity, eating, and perceived alignment with the community, work, and personal purposes. Interventions that assist individuals to record and track these behaviors and feelings and assist with behavior modification feedback are demonstrated to have a positive impact on well-being and improve health behaviors [14,15].

**Figure 1:**
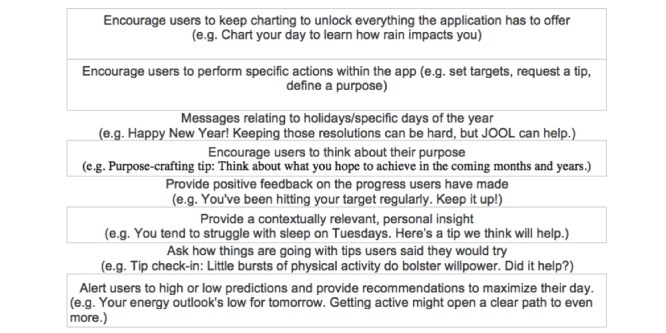
Different types of contextually tailored messages.

The app sends time-varying push notifications containing tailored health messages to provide feedback related to behavior modification and to encourage interaction with the app. The content of the feedback messages in the push notifications is drawn from a library of messages curated by JOOL to motivate, facilitate, and maintain behavior change. The content of messages is related to the purpose, energy, willpower, sleep, presence, activity, eating well, and creativity topics. The messages were created via tailoring strategies, are personalized, and the content was organized by types to both enhance message processing and interaction with the app [38] (see Figure 1). JOOL uses the answers from the self-monitoring along with environmental information (day of the week, temperature, and weather) to tailor the messages. Furthermore, to increase users’ attention, interest, and motivation to process information, the feedback messages offered in the push notifications were sent in a context that is meaningful to the recipient. To contextually tailor, first, there was a determination of the user’s context at the selected time point when the app is programmed to send a message. The context is determined by the user’s current and past data from the self-monitoring, other app usage, and environmental data such as the time of day and day of week. Next, the tailoring algorithm identified a subset of messages from the library that are meaningful to the users’ context at the decision time and randomly selects one of the messages to send to the user. For example, a user whose self-monitoring data indicate low energy is more likely to receive a message with a tip: “(...) Setting aside some time for meditation might give you more energy”; if the self-monitoring data includes reports of low willpower, the user might be sent a message such as: “(...) Little bursts of physical activity do bolster willpower.”

When a user opens the app, either prompted or unprompted, they are always required to first complete the self-monitoring intervention activity in the app. Effective engagement in response to a push notification was operationalized as interacting with the self-monitoring intervention activity in the app within the next 24 hours.

### Microrandomized Trial Design

**Figure 2:**
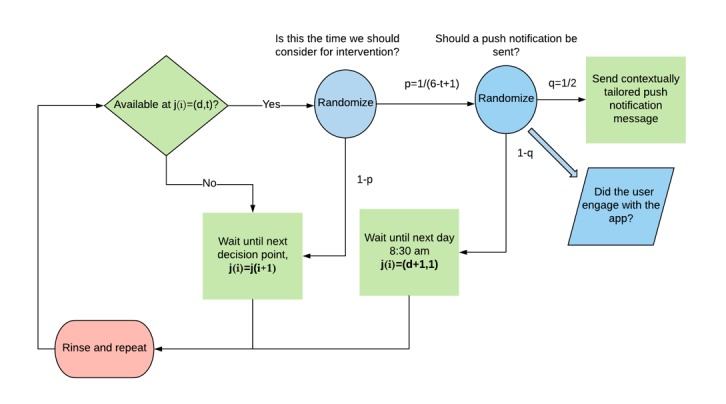
Micro-randomized trial design in JOOL app. Each decision point j (i)=(d, t) where i=1, 2,…,534, corresponding to a time of day t=1,2,…,6; 8:30 am, 12:30 pm, 5:30 pm, 6:30 pm, 7:30 pm, 8:30 pm with in a day d=1, 2, …, 89.

The implementation of the MRT design for sending push notifications in the app is shown in Figure 2. Push notifications could be sent at 1 of 6 chosen time points throughout the day, and a user could either receive or not receive a push notification at a chosen time point. The insights JOOL Health has on its user group, who are primarily office workers in a 9 to 5 work environment, informed which time points are appropriate for sending prompts. During a typical day, these time points correspond to contexts such as before work (8:30 am), during lunch (12:30 pm), early evening at home (5:30 pm, 6:30 pm, and 7:30 pm), and just before bed (8:30 pm). These are times when an office worker is likely to be less busy and thus more receptive. As prior research suggests engagement typically occurs during nonworking hours, more time points outside work hours were included [39,40]. Multiple convenient time points were chosen to ensure the timing of prompts was uniformly distributed throughout the day. Specifically, at each time point on each day, a user is first classified as being available or unavailable to receive push notifications. Unavailable users advance to the next of the 6 time points on the same day at which time their availability will be assessed again. When a user was available at a selected time point, they were randomized to either consider that time point for a push notification randomization or to advance to the next time point. At each considered time point, users were randomized to either receive or not receive a push notification containing a tailored health message with a 50% probability. Once a time point is considered, the user is then considered unavailable for the remainder of the day.

### Data Gathering Process

Microrandomizations are a standard part of the JOOL app’s quality improvement process. The MRT being reported in this paper, in particular, is part of the JOOL’s effort to improve the quality of JOOL’s “push notifications” feature. This quality improvement trial was rolled out to users in March 2017. All individuals who had the app installed on their phone and had push notifications enabled between March 2017, when the randomization software was rolled out, and August 2017 were included in this study. Users who did not use the app or used it just once after downloading the app were still included if they met the above eligibility criteria. Users who disabled push notification during the study were considered eligible until the next decision in the trial and then unavailable unless and until they re-enabled the push notifications.

A collaboration agreement was established between researchers and JOOL Health Inc. to undertake this study. The design of this study and analysis processes were carried out according to terms of service and privacy policy statements in the JOOL app, consented by the users when they created their account. For this study, a limited, nonidentifiable dataset was made available to fit the preplanned analytic models. The dataset included the MRT details and information about how and when users interacted with the app but did not include any of the details the users entered in the app. The data made available for analysis were anonymous, nonidentifiable, and housed only on JOOL Health’s servers. Institutional review board letters from both the University of Michigan and Flinders University are available.

### Outcome Measure

For all aims, the outcome is user engagement with the app, which we operationalize as whether or not the user charts in the app over the next 24 hours (as this is the first and most important interaction a user engages in after opening the app); this is a binary, longitudinal outcome. A total of 24 hours was allowed after a push notification for a user to respond because the self-monitoring is designed to be done daily; the self-monitoring questions concern behaviors and feelings over the prior 24-hour period. The primary aim tests whether there is an average effect of sending a push notification containing tailored health message versus not sending a push notification. This *main effect* is an average effect over time and over any other baseline or time-varying (eg, contextual) characteristics of the user. The secondary aims 1 and 2 focus on examining whether the effect of sending the push notification with a tailored health message differs by time (we examine week-of-study and weekdays vs weekends).

### Availability

To mitigate the risk of users either turning off notifications or deleting the app due to receiving too many push notifications, users were classified as either “available” or “unavailable” at each time point, and only those time points when users were “available” were considered for push notification decision. Several rules were applied to determine availability. First, a user is “available” to receive only 1 push notification decision in a day, so after a push notification decision was made at a time point, users were classified as “not available” for consideration at subsequent time points in that day. Second, during weekends, users were considered as “unavailable” before noon. Finally, frequent prompts could potentially alienate inactive users, and result in them either disabling notifications or uninstalling the app [20,40]. To mitigate this risk, a user was also considered either “available” or “unavailable” on a day based on their longitudinal disengagement, classified as the number of days of inactivity with the app. Users who used the app less frequently were “unavailable” to receive push notifications on a greater number of days compared with those who used the app more often. JOOL identified 4 distinct types of app users based on the clusters observed within the entire population of app users’ inactivity patterns (see Table 1). The majority of individuals who stopped using the app were clustered within 0 to 9 days, followed by 10 to 29 days, and the remainder in 30+ days. As shown in Table 1, as the number of inactive days with the app increased, the number of “unavailable” days increased. This corresponds to the decrease in frequency of prompts received as users become inactive over longer periods. Users who stopped using the app recently (2 to 9 days), or in the early phases of disengagement, were “unavailable” for 2 days after a previous push notification decision. On the other hand, users who were inactive over 30 days were “unavailable” for 15 days after a previous notification decision. Similarly, during the first 2 days when the majority of users are most active with the app, users received prompts less frequently as they are already motivated and interacting with the app.

### Data Analytic Plan

To analyze the data, we used a generalization of log-linear regression analysis specifically developed to ensure unbiased estimation of the causal effects of a time-varying treatment (ie, sending a push notification vs not) on a time-varying outcome (ie, charting over the next 24 hours) in mHealth settings. The method is a generalization of the approaches described in Boruvka et al (2016) and Dempsey et al (2017), with the use of a log-link function to accommodate the binary outcome [41,42]. These analyses pool time-varying, longitudinal data across all study participants. A separate analysis was conducted for each aim; each analysis involved prespecifying 2 sets of covariates before conducting the analyses. The first set of covariates, *X*, is used to examine moderation of the causal effect of sending a push notification with a tailored health message versus not sending a push notification (in moments of availability). The second set of control covariates (which can be time-varying) is a set of covariates that are expected to be highly correlated with app use in next 24 hours; these covariates are chosen to reduce the noise (ie, increase statistical efficiency) when assessing the effect of the push notification with a tailored health message versus none.

The causal effect is expressed on the “risk-ratio” scale, that is, on a scale that measures the probability (“risk”) of completing the monitoring activity in the next 24 hours when a push notification is sent in a moment of availability, divided by the probability of completing the monitoring activity in the next 24 hours when a push is not sent in a moment of availability. If sending a push notification has a causal effect on the probability of completing the monitoring activity in the next 24 hours, the risk ratio will be different from 1. If sending a push increases the probability of completing the monitoring activity in the next 24 hours, the risk ratio will be greater than 1. Specifically, for each analysis, we modeled the log of the risk ratio linearly in *X*, using *X*^*T*^ beta, where the dimension of the unknown vector of parameters beta is the same as the number of covariates in *X*.

Table 2 provides the covariates in *X* corresponding to each aim and the hypothesis test corresponding to each aim. The covariate *week in study* is coded as 0 for the first week, 1 for the second week, and so on up to 12 for the final week of the study. The covariate *which day* is a binary variable. It has a value of 1 when the decision time is on a Monday to Friday, or 0 when the decision time is on a Saturday or Sunday. Each of the 3 preplanned hypothesis tests used a Wald statistic to test the null hypothesis that all the terms listed in column 3 of Table 1 are 0. For the primary and secondary aims, we set the type-1 error to 5%. All SEs were adjusted for within-person correlation in the binary outcome over time. We also reported estimates (and a 95% CI) of the average causal effect of sending a push notification (vs not) on the risk-ratio scale.

In the exploratory aim, we explored the effects of the push notification with a tailored health message by the time of day within weekday (vs weekend). Here, the time-of-day covariate has 6 possible levels corresponding to 6 possible decision points at which a push notification could be sent within a day: 8:30 am, 12:30 pm, 5:30 pm, 6:30 pm, 7:30 pm, and 8:30 pm. As these are exploratory analyses, we did not conduct hypothesis tests (ie, no *P* values will be reported for the exploratory aim). Instead, for these exploratory analyses, we provided plots of the model-based estimates of the effect of the push notification with a tailored health message versus no push notification (on the log of the risk-ratio scale) across different levels of the *X* covariates and report point-wise 90% CI around these estimates.

**Table 1:** Relationship between engagement patterns and push notification frequency.

Number of days since user last engaged with the app	Number of days to wait before sending a push notification (frequency of notification)
<2	3 (twice a week)
2-9	2 (2-3 times a week)
10-29	6 (weekly)
30+	15 (fortnightly)

**Table 2:** Covariates used to model the causal effect of sending a prompt versus not for each of the three aims.

Aim	Covariates, *X*	Hypothesis test
Primary aim	Intercept	Intercept
Secondary aim 1	Intercept (week in study)	Week in study
Secondary aim 2	Intercept, day (weekday or weekend)	Day (weekday or weekend)

For the primary and both the secondary aims, the following 5 control covariates were used: (1) *week in study*; (2) *time since last chart*; (3) *pushed indicator*, an indicator for whether a push has been sent in the past (once a push is sent, this indicator has a value of 1.0 for all remaining time points, otherwise it has a value of 0); (4) *push success ratio*, that is, total number of charts within 24 hours of receiving a push notification any time in the past divided by the total number of push notification sent any time in the past (note that *push success ratio* is nested within *pushed indicator*, ie, if no push notifications are sent in the past, the value is 0); and (5) *has charted 10 time* s, that is, whether the user has charted at least 10 times, which corresponds to the number of times a user must engage to unlock insights feature within the app. For the secondary aim 2, we additionally adjusted for *which day* (weekday vs weekend) as a control covariate.

## Results

### Participant Characteristics

During the study period between March 2017 and August 2017, a total of 3300 users had the app installed on their phone, but 61.96% (2045/3300) of the users did not have push notifications enabled and were thus excluded from this study. The deidentified dataset analyzed in this study contained records from each of the 1255 eligible app users across 534 decision points (6 times per day over 89 days).

**Figure 3:**
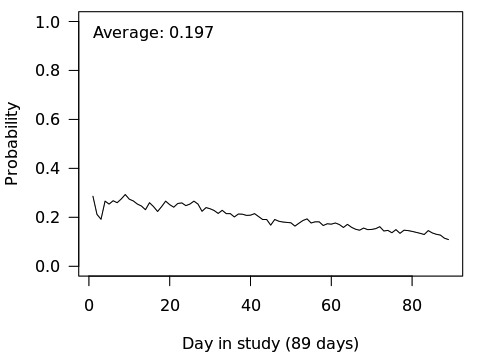
Percentage of individuals available by day in study.

Among the study sample, 63.97% (790/1235) were females, 28.86% (357/1237) were aged under 30 years, 42.44% (525/1237) were aged between 30 and 50 years, and the remaining 28.70% (355/1237) were older than 50 years. Using a body mass index cut-off of 25 or higher, 52.88% (652/1233) of the participants were either overweight or obese. Figure 3 shows the percentage of individuals available by day in the study. On an average, over the duration of the study, JOOL app users were available approximately 20% of the time. The results for each of the aims are presented below.

### Primary Aim Analysis

There is sufficient evidence to reject the primary aim null hypothesis that states there is no effect of the push notification with a tailored health message (*P*<.05, Table 3). On the basis of the results of this analysis, it is estimated that on an average, individuals are 3.9% more likely to chart in the next 24 hours when a notification with a tailored health message is sent versus when a push notification is not sent (95% CI 1.01 to 1.08).

### Secondary Aim 1 Analysis

**Figure 4:**
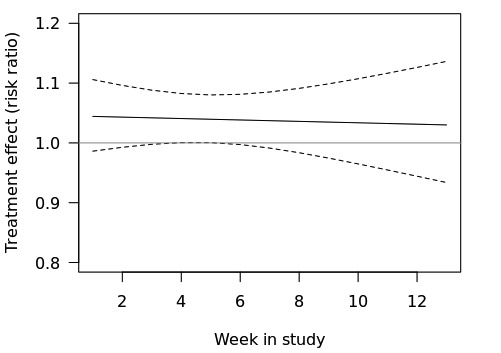
Effects of push notification over course of the trial.

On the basis of the results of this analysis (Table 4), there is insufficient evidence to reject the null hypothesis that the effect of a push notification containing a tailored health message versus not pushing a notification does not differ by week in study (*P*=.84). Figure 4 shows the estimated effects by week in study on the log risk-ratio scale (left) and risk-ratio scale (right) based on this model.

**Table 3:** Overall effects of the push notification with a tailored health message (primary aim).

Effect		Coefficient	SE	95% CI	*P* value
**Causal**					
	Decision to push (=yes)	0.04	0.02	0 to 0.08	.047
**Control covariates**					
	Intercept	−0.32	0.03	−0.38 to −0.26	
	Week in study	0.00	0.01	−0.01 to 0.01	
	Days since chart	−0.22	0.05	−0.31 to −0.13	
	Pushed indicator	−0.91	0.12	−1.15 to −0.67	
	Pushed indicator × push success ratio	0.75	0.11	0.54 to 0.97	
	Has charted 10 times	0.16	0.05	0.07 to 0.26	

**Table 4:** Effects of push notification by week in the study (secondary aim 1).

Effect		Coefficient	SE	95% CI	*P* value
**Causal**					
	Decision to push (=yes)	0.04	0.03	−0.01 to 0.10	.14
	Decision to push (=yes) x week in study	0	0.01	−0.01 to 0.01	.84
**Control covariates**					
	Intercept	−0.32	0.03	−0.38 to −0.26	
	Week in study	0	0.01	−0.01 to 0.01	
	Days since chart	−0.22	0.05	−0.31 to −0.13	
	Pushed indicator	−0.91	0.12	−1.15 to −0.67	
	Pushed indicator x success ratio	0.75	0.11	0.54 to 0.97	
	Has charted 10 times	0.16	0.05	0.07 to 0.26	

### Secondary Aim 2 Analysis

There is insufficient evidence to reject the null hypothesis that the weekend and weekday effects are not different from each other (*P*=.18, Table 5). However, the effect of a push notification with a tailored health message versus no push notification is estimated to be somewhat larger on weekends than on weekdays. Specifically, it is estimated that, *on weekends*, individuals are 8.7% more likely to chart in the next 24 hours when pushed a tailored health message versus when not pushed (95% CI 1.01 to 1.17). Whereas, *on weekdays*, individuals are 2.5% more likely to chart in the next 24 hours when pushed a tailored health message versus when not pushed (95% CI 0.98 to 1.07).

**Table 5:** Effects of push notification by weekend versus weekday (secondary aim 2).

Effect		Coefficient	SE	95% CI	*P* value
**Causal**					
	Decision to push (=yes)	0.084	0.04	0.007 to 0.156	.03
	Decision to push (=yes) x which day	−0.059	0.044	−0.145 to 0.026	.18
**Control covariates**					
	Intercept	−0.396	0.042	−0.477 to −0.313	
	Which day	0.084	0.029	0.027 to 0.141	
	Week in study	−0.002	0.006	−0.014 to 0.009	
	Days since chart	−0.220	0.046	−0.311 to −0.129	
	Pushed indicator	−0.898	0.123	−1.140 to −0.657	
	Pushed indicator x push success ratio	0.757	0.111	−0.540 to 0.974	
	Has charted 10 times	0.164	0.049	0.068 to 0.259	

### Exploratory Aim Analysis

In this analysis, we examined the effect of a push notification containing a tailored health message versus no push notification by the time of day. There are 6 times of day: 8:30 am, 12:30 pm, 5:30 pm, 6:30 pm, 7:30 pm, and 8:30 pm; on weekends, no notifications are pushed at 8:30 am. There are 2 parts to this analysis. In part 1, we examined whether the effect of the push notification varies by the time of the day. In part 2, we examined whether the effect of the push notification varies by time of day within weekend versus weekday.

#### Part (1)

The data indicate that the largest effect of the push notification containing a tailored health message is at 12:30 pm (Figure 4). Specifically, it is estimated that when the notification is pushed at 12:30 pm (vs not pushed at 12:30 pm), individuals are 8.8% more likely to chart in the next 24 hours (90% CI 1.04 to 1.15).

#### Part (2)

**Figure 5:**
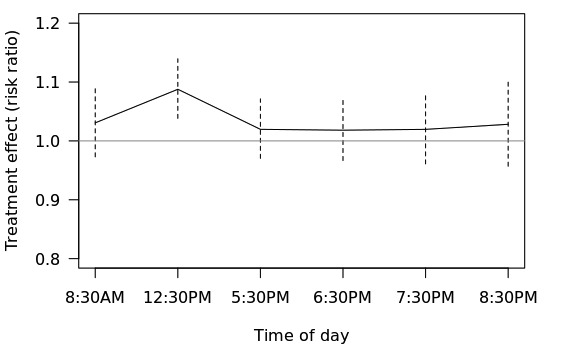
Effects of push notification over different times in a day.

**Figure 6:**
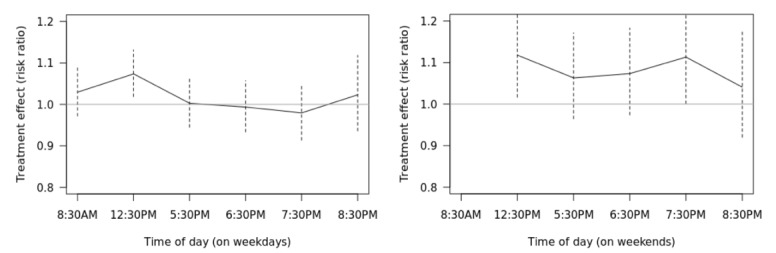
Effects of push notification over different times in a day (weekday and weekends).

During *weekdays*, the effect of the push notification was greatest at 12:30 pm (Figure 5). Specifically, it is estimated that when a tailored health message is pushed at 12:30 pm (vs not pushed at 12:30 pm), individuals are *exp* (0.071)=1.074 times as likely (ie, 7.4% as likely) to chart in the next 24 hours (90% CI 1.02 to 1.13). During *weekends*, the effects were greatest at 12:30 pm and 7:30 pm. Specifically at 12:30 pm on weekends, the risk ratio is 1.118 or 11.8% are more likely to chart in the next 24 hours (90% CI 1.02 to 1.23). The effect is similar at 7:30 pm on weekends. Figure 6 shows the effects by time of day and weekday versus weekends (with 90% confidence limits). These results are congenial with the results of the secondary aim 2 analyses, which suggested that the effects were somewhat stronger on weekends than on weekdays.

## Discussion

### Principal Findings

The results indicate that pushing a notification with a tailored health message impacts near time, proximal engagement with the self-monitoring activity in the app. The effect of the pushed notifications is sustained over time, suggesting that push notifications containing tailored health messages can attenuate the rate at which users disengage. Both the positive effect of notifications [35,43], as well as the decline in effects over time on engagement have been noted in prior research [43]. Finally, the results suggest that push notifications with a tailored health message result in higher rates of engagement during weekends and according to time at mid-day. This is contrary to the lack of effect of the timing of notifications on engagement observed in prior research [35]. Substantial differences in trial design and relatively small sample sizes may explain why these effects were not observed in previous studies.

### Implications

The findings suggest that users are more likely to engage with the app in the 24 hours following a push notification containing a tailored health message compared with no push notification. However, the effects are relatively small, with only a 3.9% greater likelihood of engaging with the app in the next 24 hours. The likelihood of users viewing the notifications within 24 hours is very high. Previously, it has been noted that the probability of users clicking a notification increases from 50% within 30 seconds to 83% in 5 min [24]. This suggests that prompts would have captured users’ attention most of the time, but their attention would have translated to engagement with the self-monitoring activity in the app less frequently. On the other hand, even small effects on engagement can be of substantial benefit at the population level, given the scalability of app-based resources. Similarly, the effects may be small because not all push notifications, even when messages are contextually tailored and personalized, are likely to be persuasive to all users at all times. In the design of this study, push notifications were sent at different times and days of the week when users are likely to be in different contexts. As a result, over the course of the trial, some users could have responded fewer times to prompts if they had received them in certain contexts where receptiveness to interruptions is low. During such contexts, particularly if engaged in a cognitively demanding activity, they may be less likely to pay attention to notifications. In fact, the results from the exploratory aim suggest that effects vary by time of the day and day of the week. In general, the largest effect occurs when the notification is pushed at 12:30 pm—users are 8.8% more likely to chart in the next 24 hours. During the weekends, the largest effects occur at 2 time points (12:30 pm and 7:30 pm). Specifically at 12:30 pm on weekends, users are 11.8% more likely to chart in the next 24 hours. The effect is similar at 7:30 pm on weekends. These findings suggest that sending push notifications with contextually tailored messages over time, particularly if sent during those contextual moments when users are most receptive, can serve as an effective strategy to maximize engagement with mHealth app interventions.

As this study population involved office workers, users who are typically at work during weekdays, it is likely that they responded more to weekend prompts because they were less busy. This was suggested by the secondary analysis where the effect of push notification versus no notification was estimated to be somewhat larger on weekends than on weekdays. The exploratory analysis suggests that on weekdays, effects are the strongest at 12:30 pm, which coincides with the time office workers generally have a lunch break. This is consistent with previous research that suggests that engagement occurs more during nonworking hours [39,40]. In summary, the findings from this study suggest that push notification prompts hold promise as an effective engagement strategy for mHealth apps, and through contextual tailoring, further advancements can be achieved in reducing user disengagement.

Tackling the problem of poor engagement is further compounded by a lack of consensus on how to operationally define engagement with an app-based health intervention. Engagement has been typically operationalized in terms of usability or usage of the intervention, along with the factors that influence these [44]. Usage can refer to the frequency or the duration of either interaction within the app, or the practice of behavioral and cognitive strategies offered by the app in the real environment. Under both scenarios, more usage, either interacting with the app more often, or practicing behaviors and cognitive strategies learned through the app more often, is viewed as better engagement. This is based on the assumption that more usage is closely related to better outcomes. However, the relationship between usage and health outcomes is weak, as supportive evidence is mostly anecdotal or correlational [45]. Instead, shifting the focus on effective engagement with the digital intervention that may or may not require sustained usage but that mediates positive behaviors is an emerging alternative [46]. In published studies of different behavioral interventions, self-monitoring is encouraged at a variety of frequencies (up to multiple times per day) [2,12,47-49]. Evidence from these studies suggests effective self-monitoring can result in a profound positive impact on health outcomes. However, the frequency of monitoring varies between individuals and over time, and as a result, it is not evident how frequently users should be encouraged to self-monitor. In fact, encouraging high rates of tracking could potentially worsen stress and cause harm [50]. Further work evaluating different self-monitoring frequencies is needed.

There are several novel aspects and strengths to this study. First, this study is designed to investigate the effect of tailored push notifications (vs no notification) on immediate proximal engagement with an mHealth app. Second, this research is naturalistic, as the study did not pay users for study involvement nor did it employ clinical staff who text, telephone, or otherwise contact users to ensure that they stay engaged in the study. There is no concept of dropout in this study. When individuals stop using the app during the trial, their outcome is recorded as disengaged in the analysis as opposed to being a dropout and excluded from the analysis. Third, the use of the MRT design to repeatedly randomize users over the course of the trial allowed us to investigate the causal effects of push notifications on proximal engagement and the real-time, real-world conditions that likely influence these proximal effects. In future, the MRT approach can also be used to adjust the probability of randomization to favor pushes in certain contexts. This experimental approach, therefore, provides the empirical evidence necessary to optimize engagement. Finally, investigating engagement in a large sample of users with an app that focuses on multiple health behaviors is another strength of this study.

### Study Limitations

Individual characteristics such as personality traits and socioeconomic status (SES) are known to influence engagement with mHealth apps, and controlling for these variables could narrow the CIs to effects of time-varying push notifications. Qualitative assessment of users’ experiences with push notifications would have offered greater insights into engagement, but as the study was constrained by data already collected within the app, such measures were not available for inclusion in our analysis. However, as noted earlier, randomizing the decision to send a push notification at a point in time ensures that within-user (eg, mood, location) and between-user (eg, personality traits, SES) factors contributing to the day-to-day variations in engagement are balanced across conditions.

Another limitation relates to the timescale used to define the proximal outcome in this study. Specifically, the proximal outcome was whether or not the individual engaged (ie, completed charting) with the app within 24 hours. A more sensitive proximal outcome measure might enable investigating the more immediate effects of prompts on engagement, potentially yielding larger effects than those observed in this study. Within these limitations, this study provides a first step to understanding whether and under what conditions push notifications promote proximal engagement in mHealth.

### Conclusions

Research into approaches for optimizing engagement with mHealth interventions is warranted and potentially valuable to the public’s health. Findings from this study suggest that push notifications can indeed influence engagement with a health app. Moreover, the results suggest that engagement effects are sustained over time but that the effect is different across contexts such as the time of day and day of week. On the basis of these results, mobile app developers are advised to incorporate push notifications as an engagement strategy and to pay attention to when prompts are sent and the types of prompts that are sent. Finally, the study offers an innovative trial design to optimize push notification delivery in mHealth apps. This approach can be incorporated into the structure of real-world apps.

Future research in this area should further investigate the contexts in which users respond to prompts and to use designs such as MRT to examine how various push-based interventions influence engagement within these contexts.

## Abbreviations

NHS: National Health Service
mHealth: mobile health
MRT: microrandomized trial
